# Chronic Styrene Exposure Causes Oxidative Stress, Neuroinflammation, and Hippocampal Memory Dysfunction via NLRP3 Inflammasome Activation

**DOI:** 10.1007/s12035-025-05472-6

**Published:** 2025-12-01

**Authors:** Raffaele Montuoro, Anna Pisani, Veronica Mohamed Hizam, Claudia Vernamonte, Giammarco Boni, Jacopo Galli, Renata Sisto, Fabiola Paciello, Anna Rita Fetoni, Claudio Grassi

**Affiliations:** 1 Department of Head and Neck Surgery, Università Cattolica del Sacro Cuore, Roma, Italy; 2 Department of Neuroscience, Unit of Audiology, Università Degli Studi Di Napoli Federico II, Naples, Italy; 3 Department of Neuroscience, Università Cattolica del Sacro Cuore, 00168 Rome, Italy; 4 Fondazione Policlinico Universitario A. Gemelli IRCCS, 00168 Rome, Italy; 5 Department of Occupational and Environmental Medicine, Epidemiology and Hygiene, Italian Workers’ Compensation Authority (INAIL), Monte Porzio Catone (RM), Italy

**Keywords:** Neurotoxicity, Cognitive decline, Oxidative stress, Microglia activation, Inflammation, Synaptic dysfunctions

## Abstract

Several studies focused on the molecular mechanisms linking oxidative stress and inflammation, due to their crucial role in different pathological conditions, including neurodegenerative disorders. Here, we studied the link between redox unbalance and neuroinflammation in a model of styrene-induced toxicity in the hippocampus. We used a rat model of chronic styrene exposure to assess its impact on hippocampal function. Behavioral tests were performed to evaluate recognition and spatial memory. Molecular and histological analyses were carried out to measure ROS production, inflammatory markers (COX-2, TNF-α, IL-1β), and immunoreactivity for IBA-1 and GFAP. Synaptic alterations, activation of pro-apoptotic pathways, and NLRP3 inflammasome levels were also evaluated. We found that toxic exposure to styrene can affect both recognition and spatial memory by causing functional, morphological, and molecular alterations in the hippocampus. Indeed, we observed increased ROS production and elevated levels of inflammatory markers, as well as increased IBA-1 and GFAP immunoreactivity, suggesting an activation of the immune system with the involvement of microglia and astrocytes in the hippocampus of styrene-treated animals. These findings were associated with molecular and structural synaptic alterations and activation of pro-apoptotic pathways. Moreover, we pointed out the possible involvement of inflammasome activation in mediating oxidative-inflammatory damage, and we documented an increased level of NLRP3 inflammasome after styrene exposure. Our data provide novel experimental evidence of styrene-induced memory dysfunctions, demonstrating that exposure to this toxic compound can impinge on the hippocampus through a mechanism involving the ROS-driven inflammation, activating the NLRP3 inflammasome axis. From a translation point of view, our results indicate that styrene exposure can be a high-risk factor for developing cognitive deficits and suggest considering the ROS/NLRP3 pathway as a target to prevent/attenuate neurotoxicity.

**Supplementary Information:**

The online version contains supplementary material available at 10.1007/s12035-025-05472-6.

## Introduction

Literature evidence supports the contribution of reactive oxygen species (ROS) to inflammatory processes [1, 2]. Indeed, increased ROS leads to the recruitment of inflammatory cells, including macrophages and neutrophils, with the consequent production of inflammatory cytokines, allowing the clearance of the pathogen responsible for tissue insult. Several reports support a positive feedback loop between oxidative stress and inflammation, showing that signaling pathways involving major inflammatory agents, such as NF-κB and TNF-α, are targets of ROS and, in turn, can influence the cell redox state [3, 4]. Therefore, many chronic diseases related to inflammation are characterized by alterations in the cellular redox state [5, 6]. Recently, inflammasomes have been proposed to mediate the link between oxidative stress and inflammation [7]. Inflammasomes are molecular complexes functioning as sentinels of innate immunity by detecting pathogen-associated molecular patterns (PAMPs) or endogenous danger-associated molecular patterns (DAMPs) [8]. When activated, these complexes initiate the maturation of proinflammatory cytokines, such as interleukin-1β (IL-1β), to promote innate immune defenses [9]. Among numerous types of inflammasomes [10], the NLR family pyrin domain-containing 3 (NLRP3) is the most comprehensively studied and is associated with a wide range of diseases, including neurodegenerative disorders [11]. Several studies have suggested that ROS production engages the NLRP3 inflammasome (rev in [7]. NLRP3 agonists induce ROS production, whereas antioxidant scavengers can suppress inflammasome activation [12–14]. In contrast, NLRP3 inflammasome-driven inflammation recruits inflammatory cells, including macrophages and neutrophils, which in turn cause ROS production [2, 15]. Considering that several studies have reported the role of the NLRP3 inflammasome in drug-induced toxicity [16, 17], we wondered whether ROS/inflammasome signaling could be involved in the neurotoxic effect exerted by the chemical agent styrene on hippocampal functions. Styrene is an aromatic hydrocarbon that is widely used in plastic and manufacturing industries and has been classified as an ototoxic, hepatotoxic, and nephrotoxic agent [18–20]. Previous studies, including ours, have documented the molecular mechanisms underlying styrene-induced ototoxicity, demonstrating that increased oxidative stress and ROS levels are the primary causes of cell death induced by styrene exposure in the peripheral auditory organ, the cochlea [21–24]. Specifically, styrene can alter cochlear redox balance by promoting oxidative stress and affecting the endogenous antioxidant system [21]. In addition to ROS damage, increased inflammation has been found after styrene exposure, with enhanced expression of the main pro-inflammatory agents [22], and macrophage activation has been observed not only in the cochlea but also in auditory brain structures [25].

Studies in workers have reported impaired vision [26, 27], altered visual contrast sensitivity, attention and memory defects [28], decreased somatosensory function, and increased vibrotactile thresholds [29] after styrene exposure. The neurotoxic effect of polystyrene nanoplastics has been recently reported* in vivo* [30], and its cytotoxicity has been established in cortical neuron preparations [31]. However, the molecular mechanisms underlying styrene-induced neurotoxicity are not completely understood and require further investigation. Thus, in this study, we explored the neurotoxic impact of styrene *in vivo*, by investigating the effect of its chronic exposure on the hippocampus, and testing the hypothesis of a ROS/NLRP3-mediated mechanism of damage.

## Materials and Methods

### Animals

**Fig. 1:**
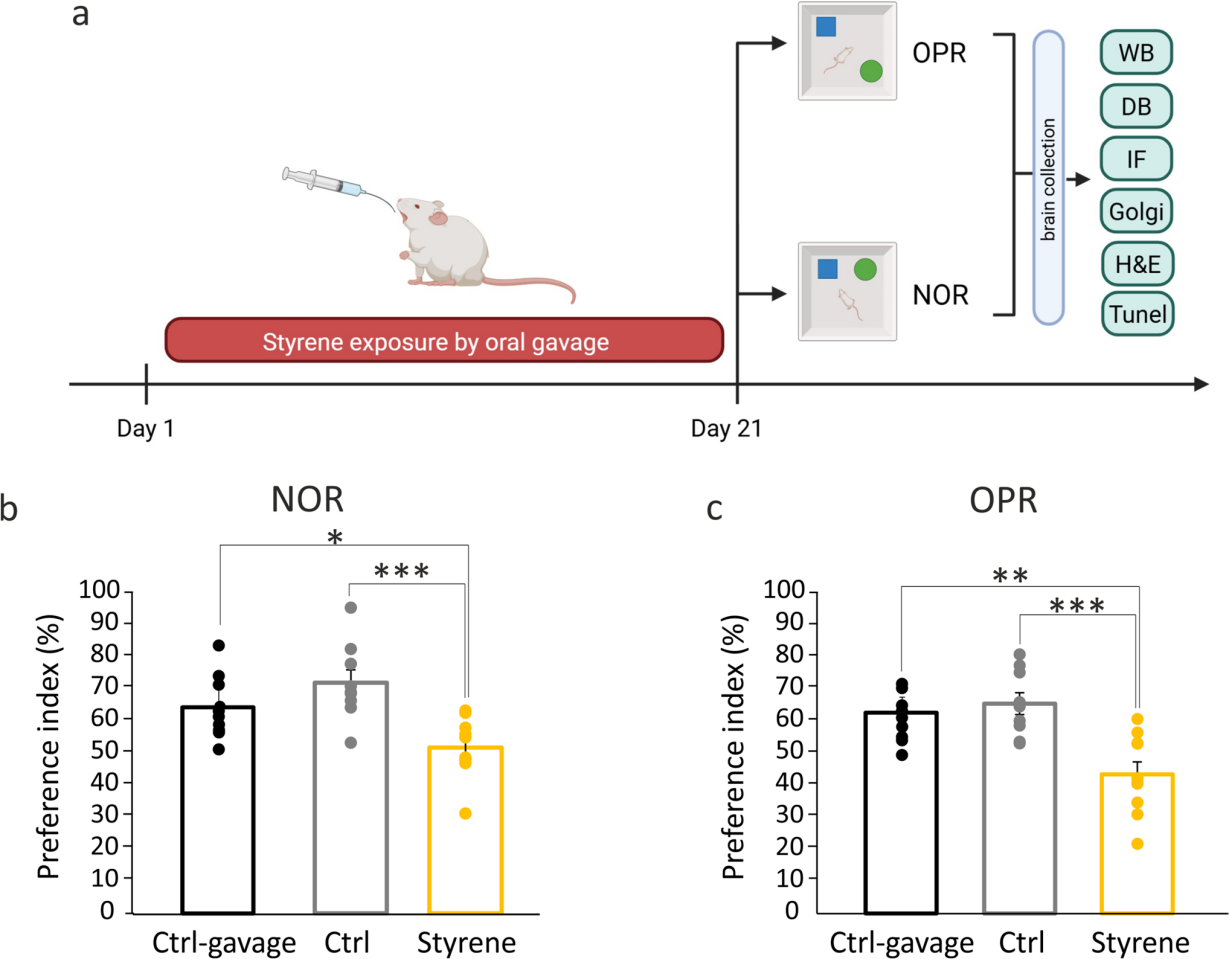
Styrene exposure causes memory deficits. **a** Schematic representation of the experimental plan. Styrene was administered daily (400 mg/kg) by oral gavage for 3 weeks (from day 1 to day 21), 5 days a week in adult Wistar rats. At the end of the treatment (day 21), recognition and spatial memory were evaluated by using novel object recognition (NOR) and object place recognition (OPR) tests. At the end of behavioral evaluations, animals were sacrificed, and hippocampal tissues were collected to perform experimental analyses. WB: western blot; DB: dot blot; IF: immunofluorescence; Golgi: Golgi–Cox staining. Created by using BioRender. **b**–**c** Graphs showing the preference index in NOR (b), and OPR (c) tests in Styrene and Ctrl groups (Ctrl *n* = 9; Ctrl-gavage *n* = 10; Styrene *n* = 10). Notably, an impairment of both recognition (b) and spatial (c) memory, were observed in animals treated with styrene (one-way ANOVA, NOR: Ctrl vs. Styrene *p* = 0.0018; Ctrl-gavage *vs. *Styrene *p* = 0.03; OPR Ctrl *vs.* Styrene *p* = 0.0006; Ctrl-gavage *vs.* Styrene *p* = 0.005). Data are expressed as mean ± SEM. Asterisks indicate significant differences among groups (**p < 0.001)

In our study, we utilized 34 male adult Wistar rats (2–3 months old) randomly divided into three groups: (1) controls (“Ctrl” group; *n* = 12); (2) styrene treated animals (“Styrene” group; *n* = 12); and (3) control group that received olive oil gavage (“Ctrl-gavage” group; *n* = 10). The rats were housed in a controlled environment with a temperature of 22–23 °C, humidity of 60 ± 5%, and a 12-h light/dark cycle, with food (Mucedola 4RF21, Italy) and water *ad libitum*. To minimize animal suffering and numbers, we adhered to the guidelines set by the European Community Council Directive of November 24, 1986 (86/609/EEC) and followed the procedures approved by the Laboratory of Animal Care and Use Committee of the Catholic University, School of Medicine of Rome, and the Italian Department of Health (*Ministero della Salute*, Prot. 1F295.116). A diagram detailing the experimental plan is shown in Fig. 1a.

### Chronic Styrene Exposure

Styrene (styrene > 99%, Sigma Corporation, product id: S4972) was mixed with olive oil as previously described [21, 22, 25]. The mixture was administered orally via gavage at a dose of 400 mg/kg per day mixed in olive oil (0.4 ml/kg body weight), 5 days a week, for a total of 3 weeks (21 days). The dosage was selected based on our previous studies demonstrating the toxic effect of styrene on the cochlea and brain structures, such as the auditory cortex [22, 25].

### Memory Tests

To study cognitive functions, we performed the novel object recognition (NOR) test to assess long-term recognition memory and the object place recognition (OPR) test to evaluate spatial memory. Behavioral testing was conducted from 9 a.m. to 4 p.m., and data analysis was performed with the aid of the automated video tracking system Any-MazeTM (Stoelting Co., Wood Dale, IL, USA). The position of the novel/displaced objects was alternated on both sides of the field to exclude a possible preference effect. Moreover, to avoid object-related preferences, the identity of the objects was counterbalanced across groups. The objects used were approximately the same size, and different shapes were used, including cubical or rectangular Lego bricks, glass jars filled with clean bedding, plastic pyramids, and spheres. Each object was secured to the arena during each phase to prevent the animals from displacing it. After each test, the objects and the apparatus were cleaned with 70% ethanol.

Additionally, to rule out deficits in locomotor activity, anxiety, or stress-related behaviors that could have affected the test results, we evaluated the distance traveled and the time spent in the corner of the open field arena during the habituation phase as indices of locomotor activity and anxiety behavior, respectively.

#### Novel Object Recognition Test

Briefly, as described in [32], the test was divided into three different sessions: habituation, training, and a test phase, each divided by 24 h. In the habituation session (first day), the animals were introduced to the test arena (90 cm × 90 cm) for 10 min. In all the described phases, we placed various cues on the walls to serve as spatial reference points. In the training session (second day), rats were allowed to explore for 10 min two identical objects located symmetrically in the center of the arena. During the third day, in the NOR test, one of the familiar objects was replaced with a new object, and the animals were given 5 min to explore the arena. After scoring for time spent exploring the objects during the NOR test phases, a preference index was calculated as the ratio of the time spent exploring the novel/displaced objects to the time spent exploring both objects.

#### Object Place Recognition Test

OPR test was performed in the same arena (90 cm × 90 cm) used for NOR. Similarly to NOR, the test was divided into habituation, training, and a test phase. In the habituation session (first day), the animals were introduced to the test arena for 10 min. In the training phase, rats were allowed to explore for 10 min two identical objects located symmetrically in the center of the arena, and in the test phase, one of the objects (specifically the old object in the NOR test) was moved toward one of the borders of the arena, and animals were allowed to explore for 5 min. After scoring for time spent exploring the objects during the OPR test phases, a preference index was calculated as the ratio of the time spent exploring the novel/displaced objects to the time spent exploring both objects.

### Immunofluorescence Staining

Once behavioral testing, animals were sacrificed, and the brains were promptly removed after transcranial perfusion with 0.9% NaCl, and subsequently with paraformaldehyde (PFA) solution. Samples were then fixed with 4% PFA in PBS at 4 °C and a pH of 7.4. To perform immunofluorescence analysis, 40-µm-thick coronal brain sections were obtained by using a cryostat (SLEE Medical GmbH, Germany). To evaluate ROS amount, slices were incubated with 1 µM DHE in PBS for 30 min at 37 °C and then coverslipped with an antifade medium. For 8-isoprostane, IBA-1, and GFAP immunostaining, slides were incubated in a blocking solution containing 1% fatty acid-free bovine serum albumin (BSA), 0.5% Triton X-100, and 10% normal goat serum in PBS for 1 h at room temperature (RT). Next, brain sections were incubated overnight at 4 °C with a solution containing primary antibodies (see Table S1). All slides were then washed twice in PBS and incubated at RT for 2 h in a solution with labeled conjugated secondary antibodies (Table S1). Following another PBS wash, the samples were incubated with DAPI for 20 min in the dark at RT and coverslipped with an antifade medium (Table S1). Fluorescent images were captured using a confocal laser microscope (Nikon Ti-E; Confocal Head A1 MP, Tokyo, Japan) equipped with a 20 × objective lens. ImageJ software (version 1.51 s) was used to count IBA-1-positive cells and GFAP immunoreactivity in at least three sections from four animals/groups. To ensure the accuracy of the results, control experiments were performed by randomly omitting the primary antibody incubation of tissues selected across the experimental groups (data not shown). To minimize variability related to antibody penetration, incubation time, and tissue condition, all tissues from the different groups were processed together during the procedures.

### TUNEL Assay and H&E Staining

TUNEL assay Kit (Table S1) was used to detect DNA fragmentation in the nuclei of apoptotic cells in the hippocampus. The assay was performed according to manufacturer’s instructions. All procedures were performed under dim light. Briefly, the brain slices were incubated 2 h in freshly prepared TUNEL reaction mixture. The specimens were rinsed twice in PBS and then three times in a solution containing 0.1% Triton X-100 and BSA in PBS to reduce background. Then slices were incubated with DAPI (Table S1) for 15 min at room temperature. After rinsing three times in PBS, specimens were coverslipped with an antifade medium (Table S1). Cells with intense, pink-labeled nuclei (red plus blue) were identified as apoptotic cells.

Hematoxylin and eosin (H&E) staining was performed using the H&E Staining Kit (Table S1) in brain slices from three animals/groups. A H&E kit (Table S1) was used and brain sections were processed following manufacturing instructions.

### Spine Density Evaluation

To assess the quantity of dendritic spines in neurons within the dentate gyrus (DG) region of the hippocampus, brains of four animals/group were collected following transcardiac perfusion with 0.9% NaCl and stained using the Golgi–Cox solution, based on a previously published method [25]. The spines were counted only if they protruded laterally from the neuronal dendrites, excluding those above or below the dendrite. Spine density was determined along a dendritic terminal length of approximately 20 μm. An Olympus BX63 microscope equipped with a 100 × oil immersion objective lens was used to acquire images, and the spine count was performed by using Image J software (version 1.51 s).

### Western Blot and Dot Blot

Hippocampal tissues from three or four animals/groups were homogenized in lysis buffer with 150 mM NaCl, 50 mM Tris–HCl pH 7.4, 2 mM EDTA, 1% Triton X-100, 0.1% SDS, protease inhibitor cocktail, sodium orthovanadate, and sodium fluoride. The homogenate was sonicated for 10 s “on” and 20 s “off” for three rounds using a Diagenode Bioruptor Standard water bath sonicator. The sample was then spun down at 22,000 × *g* and 4 °C. Equal amounts of protein were boiled and resolved using SDS-PAGE, as previously described [33]. For dot blotting, 5 μl of lysate (5 μg/μl) was spotted onto a TBST-prewetted nitrocellulose membrane, as described previously [33]. After the overnight incubation with a solution containing the primary antibodies (Table S1), membranes were incubated with HRP-conjugated secondary antibodies (Table S1), washed in TTBS. After, chemiluminescent substrates (Cyanagen, Bologna, BO, Italy) were used, and band density was documented and quantified using the UVItec Cambridge Alliance. The equal protein loading was verified by using GAPDH or β-actin loading control (Table S1). Ponceau S was used as the loading control for dot blotting.

### Statistical Analysis

The sample size was chosen after performing a power analysis to achieve a statistical power of 80% at a significance level of 0.05. Statistical analyses were performed in a blind manner. To perform statistical comparisons, One-way ANOVA or Student’s *t* test was employed, as specified in the main text and figure legends. The results are reported as the mean ± SEM.

## Results

### Styrene Causes Memory Impairment and Hippocampal Synaptic Alterations

To evaluate a possible neurotoxic effect of styrene on hippocampal functions, animals were daily subjected to styrene or vehicle oral gavage administration for 21 days (Fig. 1a). At the end of this treatment, recognition and spatial memory were assessed by the NOR and OPR tests respectively. In styrene-treated animals, we found a significant decrease in the preference index in both NOR and OPR tests (Fig. 1b, c) compared to both Ctrl and Ctrl-gavage groups, indicating a negative impact of styrene on recognition and spatial memory (one-way ANOVA, NOR: F_(2,26)_ = 9.308, Ctrl *vs.* Styrene, *p* = 0.0018; Ctrl-gavage *vs.* Styrene, *p* = 0.03; OPR: F_(2,26)_ = 13.20, Ctrl *vs.* Styrene *p* = 0.0006; Ctrl-gavage *vs.* Styrene *p* = 0.005). These effects of styrene on memory functions were not dependent on either stress-related behavior or locomotor activity deficits. Indeed, no significant differences in the time spent in the border of the open field arena, as well as in the total distance traveled, were found comparing styrene, Ctrl, and Ctrl-gavage groups (Fig. S1, one-way ANOVA, F_(2,26)_ = 1.88, *p* > 0.05). Finally, no significant differences in memory performance in all tests used were observed by comparing Ctrl-gavage *vs.* Ctrl group (Fig. 1a, one-way ANOVA, NOR: F_(2,26)_ = 9.308, *p* > 0.05; OPR: NOR: F_(2,26)_ = 13.20, *p* > 0.05) excluding any potential impact of gavage procedure or olive oil administration on cognitive functions. Thus, all further morphological and molecular analyses were performed on Styrene and Ctrl samples.

**Fig. 2:**
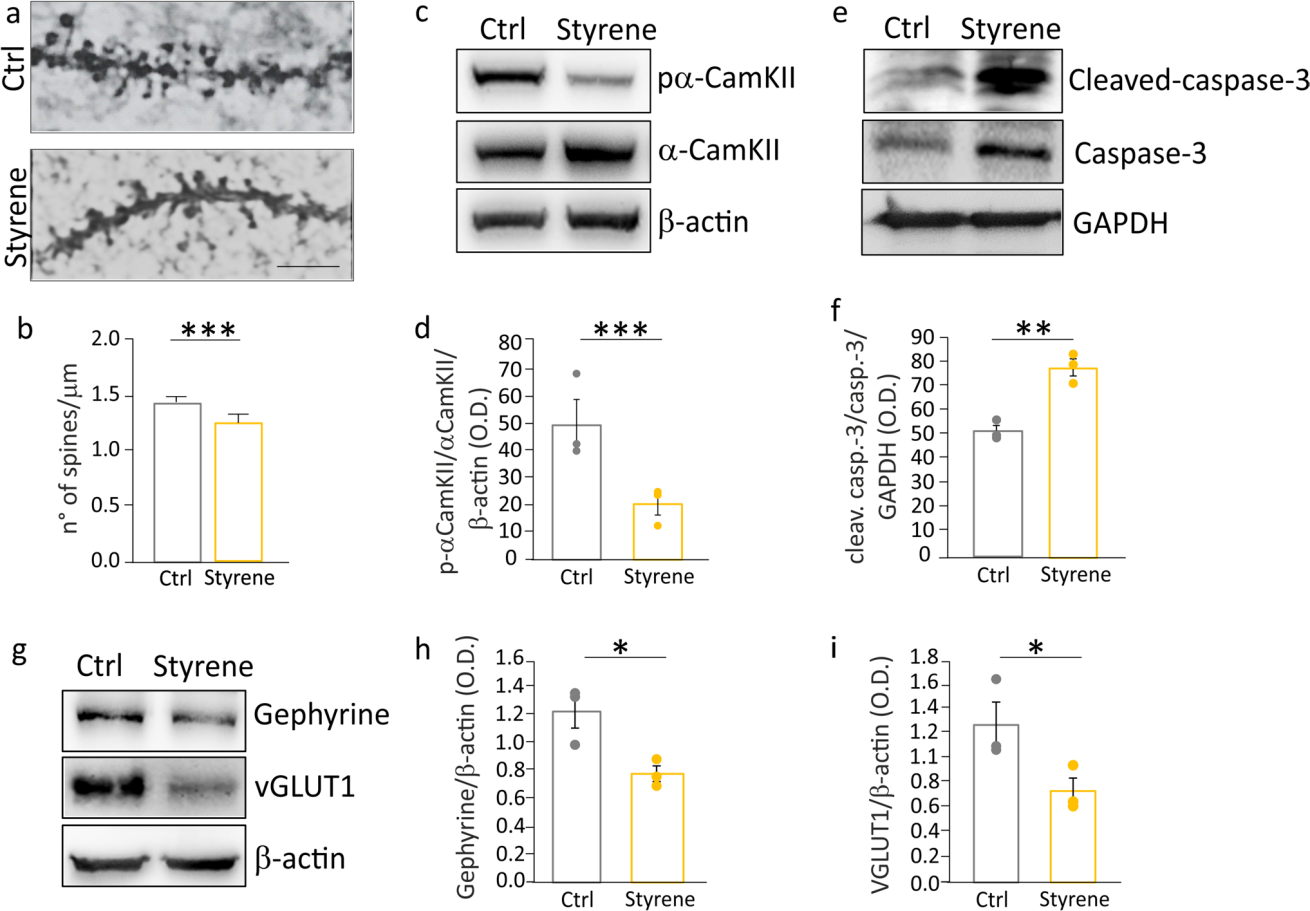
Synaptic damage in the hippocampus of styrene-treated animals. **a** Representative images of Golgi-Cox-stained dendritic segments of dentate gyrus (DG) neurons from Ctrl and Styrene animals. Scale bar: 5 μm. **b** Histograms showing dendritic spine density data (Ctrl *n* = 3, Styrene *n* = 3, Student’s *t* test, *p* = 0.026). **c**, **e**, **g** Representative western blot bands showing lower pα-CamkII^T286^ (c), higher cleaved caspase-3, (e), and reduced gephyrin and vGLUT1 (g) levels in styrene-treated animals. **d**, **f**, **h**, **i** Histograms (mean ± SEM) show densitometric analyses in all samples (*n* = 3 animals for each group; Student’s *t* test, pα-CamkIIα^T286^ *p* = 0.04; Caspase-3 *p* = 0.003; vGLUT1 *p* = 0.02; Gephyrin *p* = 0.02). Asterisks indicate statistical significance (*p < 0.05; ***p < 0.001)

Having established a detrimental action of styrene exposure on memory, we performed morphological and molecular analyses in the hippocampus of treated rats. Our Golgi-Cox staining experiments revealed a significant decrease of spine density in DG neurons of the hippocampus of styrene-treated animals with respect to controls (Fig. 2a, b; Student’s *t* test, *p* = 0.026). The reduced number of spines was also associated with a significant decrease of the phosphorylation at threonine 286 (T286) of Ca2 +/calmodulin (CaM)-dependent protein kinase II (αCAMKII) (Fig. 2c, d; Student’s *t* test, *p* = 0.04). In hippocampal extracts of styrene-treated rats, we also found a significant increase in cleaved-caspase 3 levels compared to control values (Fig. 2e, f; Student’s *t* test, *p* = 0.003), in association with an increased number of TUNEL-positive neurons in both DG and CA1 regions (Fig. S2), suggesting the activation of apoptotic processes and cell death induced by the toxic compound. To test if neuronal loss involved both inhibitory and excitatory neurons, we performed molecular analyses evaluating a marker of excitatory synapses, such as the vesicular glutamate transporter 1 (vGLUT1), and a marker of inhibitory synapses, gephyrin, a scaffold protein responsible for organizing the inhibitory postsynaptic density [34]. Our data showed a decreased level of both gephyrin (Fig. 2g, h; Student’s *t* test, *p* = 0.02), and vGLUT1 (Fig. 2g, i; Student’s t test, *p* = 0.02) in the hippocampus of styrene-treated animals respect to controls.

Finally, morphological analyses on H&E-stained sections revealed structural alterations in DG and CA1 regions of the hippocampus, with disorganization and areas of cell loss in DG, vacuolation and decreased thickness of layer of pyramidal cells in CA1 reaching two layers in some areas (Fig. S3).

Collectively, these results suggest that chronic exposure to styrene exerts a detrimental effect on hippocampal structure and function, consisting of neuronal loss, synaptic damage affecting both excitatory and inhibitory neurotransmission, and memory deficits.

### Oxidative Stress and Endogenous Antioxidant Response in the Hippocampus of Styrene-Treated Animals

**Fig. 3:**
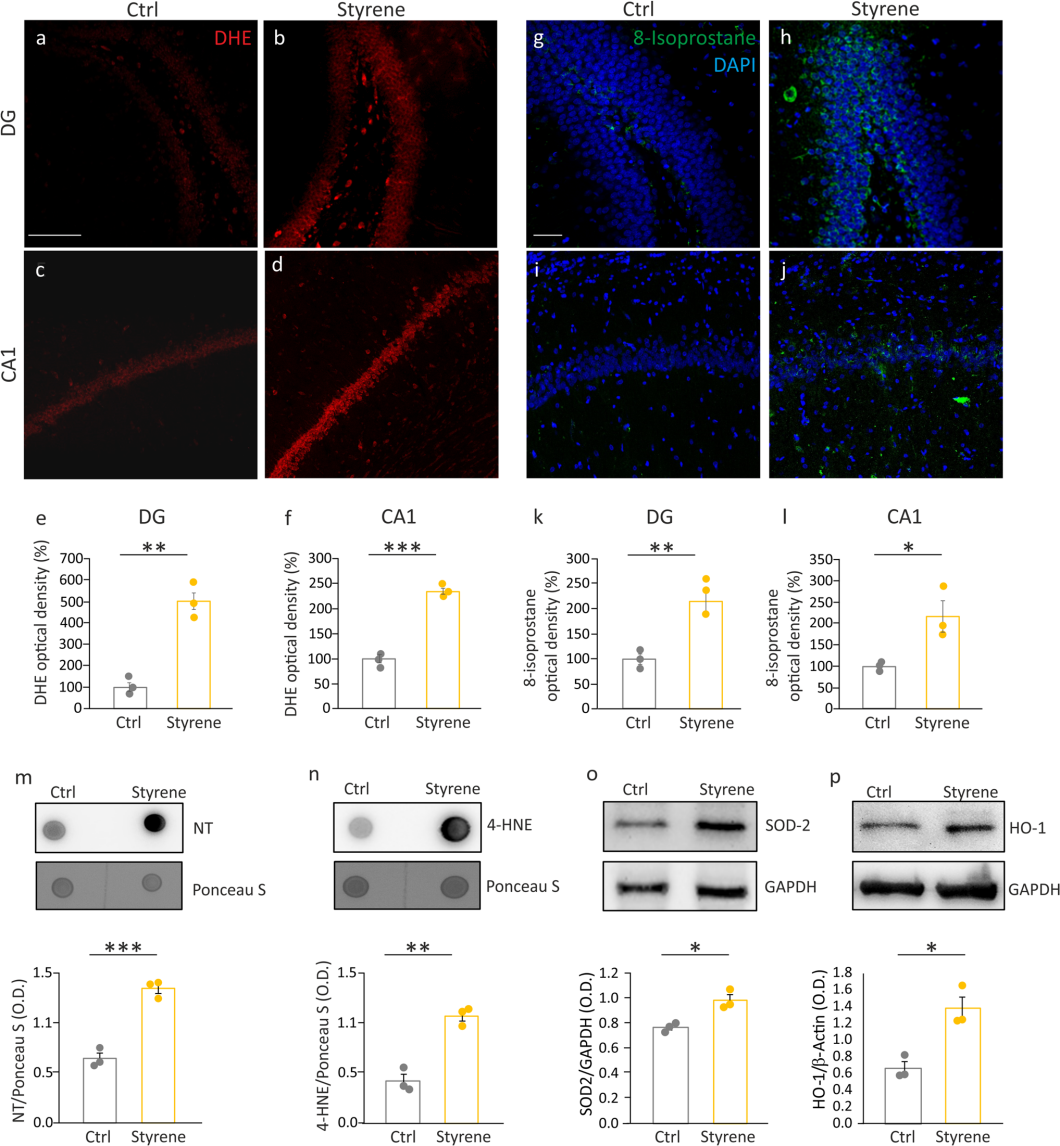
The neurotoxic effect of styrene affects hippocampal redox status. **a**–**d** Representative confocal images showing DHE fluorescence in the dentate gyrus (DG, a,b) and in CA1 (c,d) hippocampal regions of Ctrl and styrene-treated animals. **e**–**f** Histograms (mean ± SEM) show the quantification of fluorescence intensity signal normalized to control value (taken as 100%) in DG (e; Ctrl *n* = 3, Styrene *n* = 3; *p* = 0.001, Student’s *t* test) and CA1 (f; Ctrl *n* = 3, Styrene *n* = 3; *p* = 0.0002, Student’s *t* test). **g**–**j** Representative confocal images of brain sections containing hippocampus (both DG and CA1 regions) stained with 8-isoprostane as a marker of lipid peroxidation. Blue fluorescence indicates DAPI staining of cell nuclei. Scale bar: 50 µm. **k**–**l** Histograms (mean ± SEM) show the quantification of 8-isoprostane fluorescence intensity signal normalized to control value (taken as 100%) in DG (k) and CA1 (l). **m**, **n** Representative dot blot showing increased protein tyrosine nitration (NT, m) and lipid peroxidation (4-HNE, *n*) in the hippocampus of styrene-treated rats. Histograms (mean ± SEM) show the densitometric analyses (NT: Ctrl *n* = 3, Styrene *n* = 3, Student’s *t* test, *p* = 0.007; 4-HNE: Ctrl *n* = 3, Styrene *n* = 3, Student’s *t* test, *p* = 0.001). **o**, **p** Representative western blot bands indicating an increased levels of SOD-2 and HO-1 after styrene insult. Histograms (mean ± SEM) show the densitometric analyses (SOD-2: Ctrl *n* = 3, Styrene *n* = 3, Student’s *t* test, *p* = 0.01; HO-1: Ctrl *n* = 3, Styrene *n* = 3, Student’s *t* test, *p* = 0.01). *p < 0.05; **p < 0.01; ***p < 0.001

Looking for molecular mechanisms underlying hippocampal functional and synaptic alterations, we started investigating redox imbalance. Indeed, we previously demonstrated that styrene triggers oxidative stress in other brain regions, such as the auditory cortex [25], as well as in the cochlea [22]. Prompted by these data, we performed DHE analyses to detect ROS production in brain sections containing the hippocampi of styrene and Ctrl rats. Our results showed a marked increase in DHE fluorescence, indicating a rise in ROS amount in the hippocampus of styrene-treated animals, compared to controls (Fig. 3a–d), with a significant increase in fluorescence intensity in both DG and CA1 hippocampal regions (Fig. 3e–f; Student’s *t* test, DG *p* = 0.001, CA1 *p* = 0.0002). To confirm the increase of oxidative stress, we performed a dot blot to detect nitrotyrosine (NT), a marker of protein nitrosilation, representing a prominent post-translational redox modification [35]. Our dot blot analysis showed an increased NT level in hippocampal samples of animals exposed to styrene compared to controls (Fig. 3m; Student’s *t* test, *p* = 0.007). To further investigate oxidative stress, we also focused on lipid peroxidation, considering that increased ROS production can lead to peroxidation of membrane lipid bilayer [36]. Thus, we assessed lipid peroxidative damage by performing an immunofluorescence for 8-isoprostane. Similarly to what was observed for ROS production, we found a marked increase of 8-isoprostane labeling in styrene-treated samples, compared to controls (Fig. 3g–j; Student’s *t* test, DG *p* = 0.005, CA1 *p* = 0.03). The analysis of the fluorescence intensity confirmed the increased 8-isoprostane labeling in both DG and CA1 hippocampal regions of styrene-treated animals (Fig. 3k–l). Moreover, dot blot analysis also showed an increased level of 4-HNE, a well-known marker of lipid peroxidation, in styrene-treated animals (Fig. 3n; Student’s *t* test, *p* = 0.001), further confirming immunofluorescence results.

Finally, we studied the endogenous antioxidant defense system to gain insight into redox status imbalance. We focused on endogenous players of defense against free radical-induced damage, such as superoxide dismutase 2 (SOD-2) and the inducible isoform of heme oxygenase-1 (HO-1).

Our western blot analysis revealed a significant increase of SOD-2 and HO-1 levels in the hippocampus of styrene-exposed animals compared to the not-exposed group (Fig. 3o, p; Student’s *t* test, SOD-2 *p* = 0.01; HO-1 *p* = 0.01), suggesting an endogenous antioxidant response activation to face the toxic insult, although not sufficient to restore redox balance.

### Styrene Induces Inflammation and Microglia/Astrocyte Activation Triggering Inflammasome NLRP3 Pathway

**Fig. 4:**
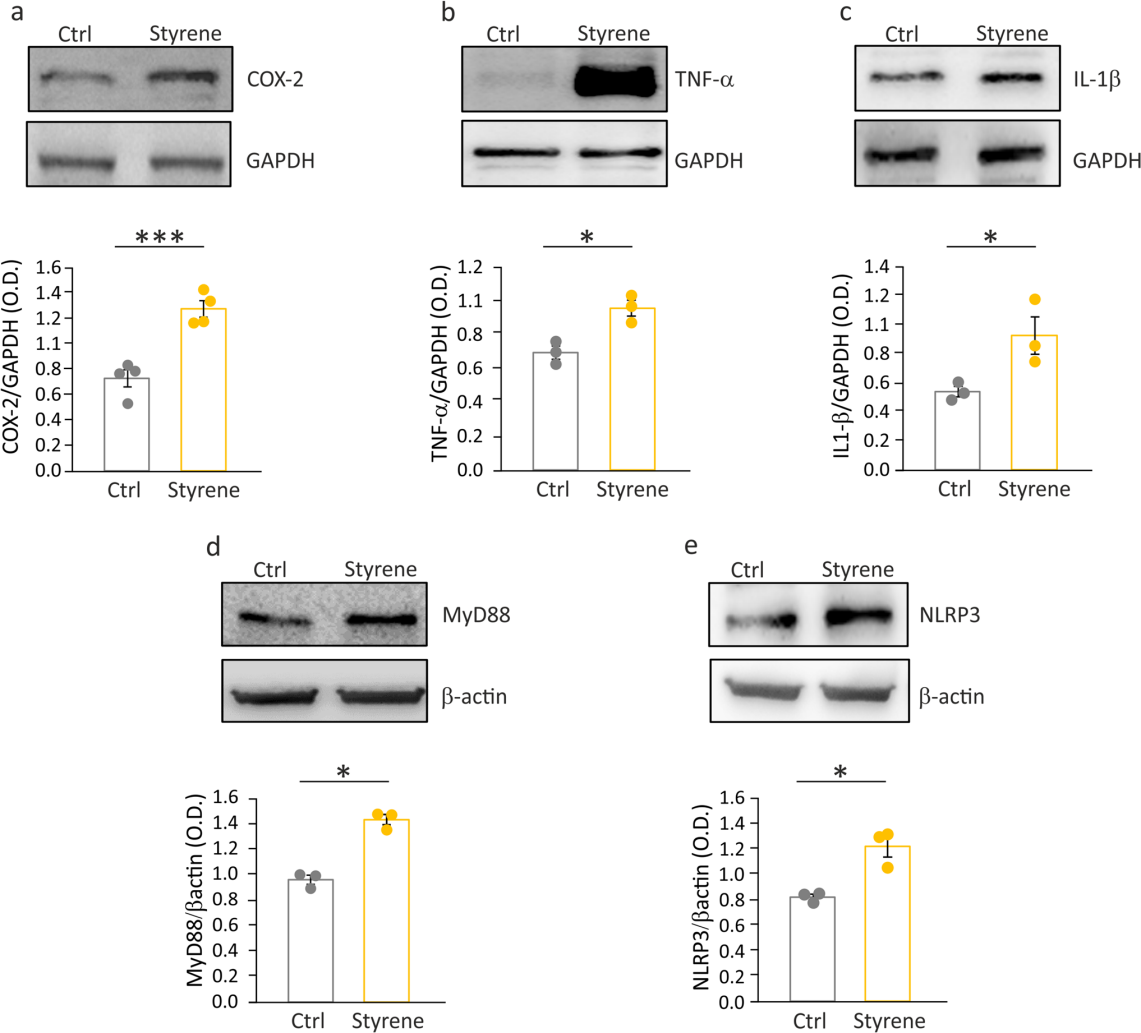
Neuroinflammation and inflammasome activation after styrene exposure. **a**–**c** Representative western blot bands showing the level of three main inflammatory markers: COX-2 (a), TNF-α (b) and IL-1β (c) in the hippocampus of control and styrene-treated animals. Histograms (mean ± SEM) show optical density values (COX-2: Ctrl *n* = 4, Styrene *n* = 4, Student’s *t* test, *p* = 0.001; TNF-α: Ctrl *n* = 3, Styrene *n* = 3, Student’s *t* test, *p* = 0.01; IL-1β: Ctrl *n* = 3, Styrene *n* = 3, Student’s *t* test, *p* = 0.04). **d**, **e** Western blot and histograms with densitometric analyses showing MyD88 and NLRP3 levels modulated by styrene exposure (MyD88: Ctrl *n* = 3, Styrene *n* = 3, Student’s *t* test, *p* = 0.0009; NLRP3: Ctrl *n* = 3, Styrene *n* = 3, Student’s *t* test, *p* = 0.01). Asterisks indicate statistical significance (*p < 0.05; **p < 0.01; ***p < 0.001)

Prior studies, including ours, provided evidence of increased inflammation associated with redox imbalance in the cochlea and brain structures (auditory cortex) after exposure to styrene [21–23, 25]. Thus, we evaluated the expression of common inflammatory markers in the hippocampus of styrene-exposed animals. Specifically, we focused on key mediators of inflammatory pathways, including cyclooxygenases-2 (COX-2), TNF-α, and IL-1β [37–39]. We found a significant increase in the levels of all inflammatory markers analyzed in styrene-treated animals compared to controls (Fig. 4a–c; Student’s *t* test, COX-2 *p* = 0.001; TNF-α *p* = 0.01; IL-1β *p* = 0.04), indicating a combined detrimental effect of oxidative stress and inflammation caused by styrene toxic insult in the hippocampus.

**Fig. 5:**
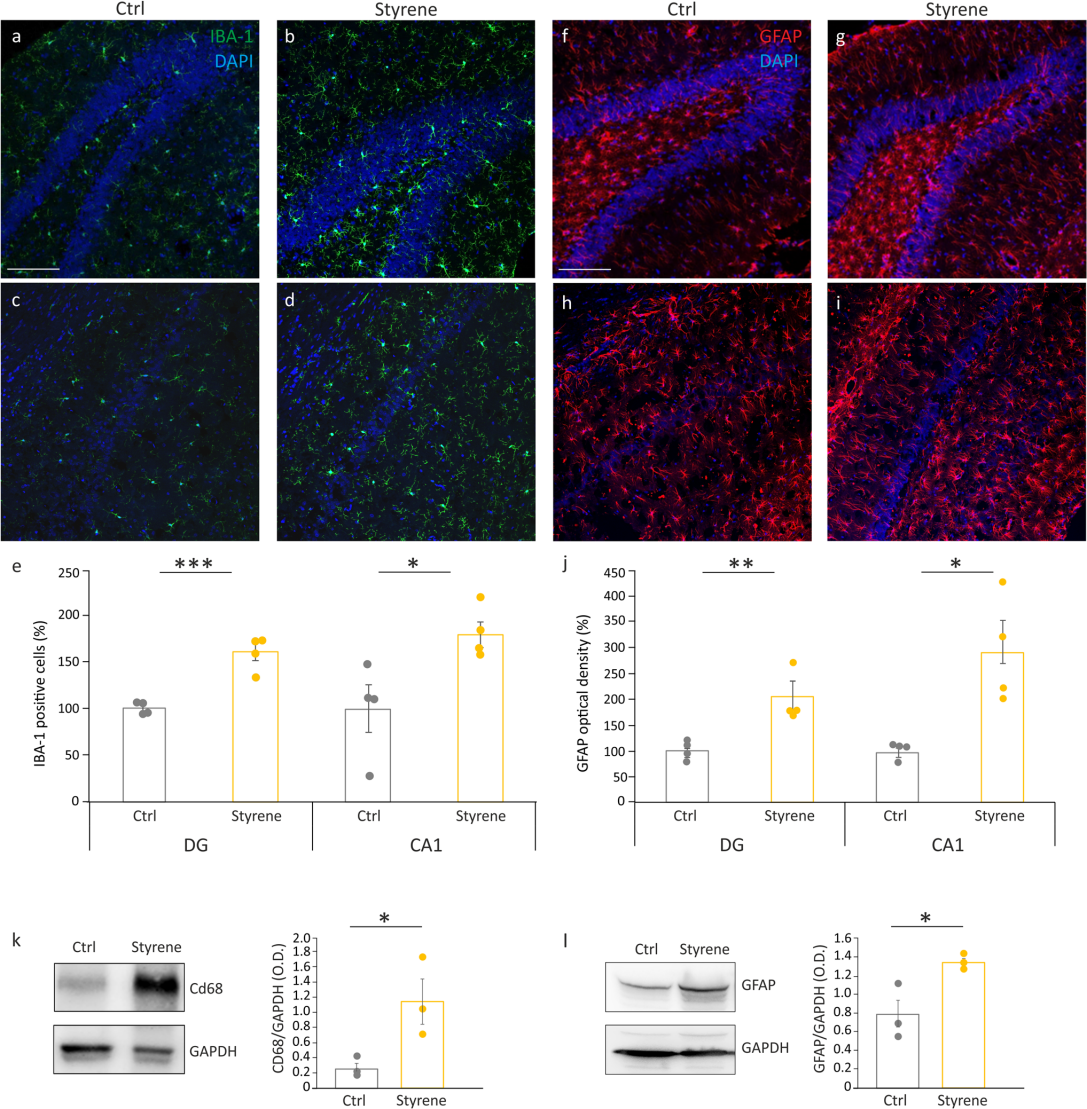
Microglia and astrocyte activation in the hippocampus of styrene-treated animals. **a**–**d** Representative images of brain coronal sections showing IBA-1 (green fluorescence) and DAPI staining (blue fluorescence) in the hippocampus (DG a-b and CA1 c,d) of control and styrene-treated animals. **e** Histogram (mean ± SEM) showing the percentage of IBA-1 positive cells normalized to control values (Ctrl *n* = 4 *vs.* Styrene *n* = 4; CA1 *p* = 0.03; DG *p* = 0.001; Student’s *t* test). **f**–**i** Representative images showing GFAP staining (red) and DAPI staining (blue) in the hippocampus (DG f-g and CA1 h-i) of control and styrene-treated animals. **j** The graph (mean ± SEM) shows GFAP optical density expressed as percentage and normalized to control values (Ctrl *n* = 4, Styrene *n* = 4; DG *p* = 0.006; CA1 *p* = 0.01, Student’s *t* test). Scale bar: 50 µm. **k**–**l** Representative western blot bands showing the level of CD68 (k) and GFAP (l) in total hippocampal lysates from control and styrene treated animals. Histograms (mean ± SEM) show optical density values (CD68: Ctrl *n* = 3, Styrene *n* = 3, Student’s *t* test, *p* = 0.04; GFAP: Ctrl *n* = 3, Styrene *n* = 3, Student’s *t* test, *p* = 0.3). Asterisks indicate significant differences between groups (***p < 0.001; *p < 0.05)

It is now recognized that inflammation induced by tissue damage is an essential mechanism of innate immune response. Thus, we performed immunofluorescence analyses to study microglia, the resident cells in the central nervous system (CNS) that can mediate immune responses [40]. Our data showed a significant increase of IBA-1 positive cells (a general marker of macrophages and microglia) both in the DG and CA1 hippocampal regions of styrene-treated animals (Fig. 5b, d) compared to controls (Fig. 5a, c), as also confirmed by IBA-1 positive cells count (Fig. 5e; Student’s *t* test, CA1 *p* = 0.03; DG *p* = 0.001). Additionally, western blot analyses showed a significant increase of CD68, the microglia phagocytic marker [25, 41], in the hippocampus of styrene-treated rats (Fig. 5k; Student’s *t* test, *p* = 0.04). We also studied astrocytes by performing GFAP labeling, and we found a significant increase of GFAP immunoreactivity in the hippocampus (both DG and CA1 regions) of animals exposed to styrene (Fig. 5f–i), confirmed by the analysis of the fluorescence intensity signal (Fig. 5j; Student’s *t* test, DG *p* = 0.006; CA1 *p* = 0.01). Finally, western blot data further demonstrate the increased GFAP level in hippocampal lysates from styrene-treated animals with respect to controls (Fig. 5l; Student’s *t* test, *p* = 0.3). Furthermore, to gain more insights into the role of the innate immune response, we assessed the levels of MyD88, which is a downstream adaptor protein of the toll-like receptors (TLR), a family of receptors involved in pathogen recognition and host defense [42]. Upon TLR activation, MyD88 is responsible for a cascade of molecular events leading to the activation of the inflammatory response [43]. Consistently, our western blot data showed increased levels of MyD88 in the hippocampus of styrene-exposed rats (Fig. 4d; Student’s *t* test, *p* = 0.0009), similar to those observed for TNF-α and IL-1β.

Finally, we focused on pyrin domain-containing protein 3 (NLRP3) inflammasome protein. Our western blot analyses showed significantly higher levels of NLRP3 in the hippocampi of styrene-treated animals (Fig. 4e; Student’s *t* test, *p* = 0.01), also consistent with the increased expression of IL-1β, a downstream target of the NLRP3 pathway [44]. Collectively, these data indicate that the styrene-damaging effect involves the inflammasome pathway, leading to increased inflammatory markers and microglia/astrocyte activation.

## Discussion

In this study, we investigated the detrimental effect of chronic styrene exposure on the hippocampus, and we explored the potential role of the NLRP3 inflammasome in the pathogenesis of styrene-induced neurotoxicity. Although the ototoxic effect of styrene is well established, the damaging impact of this volatile compound on cognitive functions and possible underlying mechanisms still needs further investigations. Here, we demonstrated that styrene administration can affect recognition and spatial memory in Wistar rats. At the molecular level, we found increased oxidative stress and altered endogenous antioxidant responses, an increase of neuroinflammatory markers, and the activation of inflammasome NLRP3 in the hippocampus. Collectively, our results provide experimental evidence supporting the toxic effect of chronic styrene exposure on cognitive functions, highlighting from a translation point of view, a possible environmental risk at the occupational level for developing dementia.

Our findings showing an altered redox balance in the hippocampus of styrene-treated animals are consistent with the free radical-induced damage suggested for styrene toxicity in different tissues, including the lung, liver, and cochlea [21, 22, 25, 45, 46]. In humans, styrene is mainly metabolized by cytochrome P450 to styrene 7,8-oxide, the principal reactive and genotoxic intermediate of styrene [47, 48]. Styrene 7,8-oxide exerts its toxicity by forming covalent adducts with DNA, RNA, and proteins [49] and has been proposed to have a direct oxidative stress effect on cells [50]. It has to be considered that the hippocampus is particularly vulnerable to oxidative stress compared to other brain regions [51], as well as to the detrimental effect of toxic agents [52]. Moreover, the imbalance of redox status and the ability of the antioxidant defense mechanisms to face oxidative insult play a key role in hippocampal-dependent memory deficits in different models of cognitive dysfunctions [53]. Together with increased oxidative stress, we also found neuronal death and altered spine density and molecular determinants of neural cell death in the hippocampus of styrene-treated animals, potentially affecting both excitatory and inhibitory neurotransmission. Memory formation in the hippocampus relies on synaptic plasticity, a mechanism depending on spine density and structural changes in dendritic spines [54], as well as on the balance between excitatory and inhibitory neurotransmission [55]. Oxidative stress and inflammation can alter neuronal dendritic structure [56], also involving the activation of glial cells. The mechanisms by which inflammation affects dendritic spines include activation of caspases via the release of ROS, which causes decreased spine density [57]. Furthermore, it has been shown that the release of IL-1β by microglia during the inflammatory process affects dendritic spines by antagonizing spinogenesis [58], and ROS production and microglial overactivation are closely interlinked [59] in animal models of toxic damage induced by environmental pollution [60]. Macrophages are known to produce ROS to counteract neurotoxic insults, and microglia have been recognized as the most important source of ROS in the CNS [61]. Bacterial endotoxins, lipopolysaccharides, and several chemical agents can stimulate mitochondrial superoxide production in microglia [62, 63]. Finally, the altered redox balance is involved in regulating microglia-mediated neuroinflammation [64]. We previously documented that styrene affects auditory function by causing oxidative stress and triggering the inflammatory response, with increased expression of the main inflammatory markers, including NF-kB, TNF-α, and IL-1β [22], and microglial activation [25]. Here, we documented a direct toxic effect of styrene in the hippocampus, and we attempted to unveil the mechanism linking ROS production, inflammation, and microglia activation by focusing on inflammasomes. The term ‘‘inflammasome’’ refers to the assembly of an intracellular multiprotein complex as a molecular platform that triggers an inflammatory response that regulates the maturation and secretion of potent pro-inflammatory cytokines, such as IL-1β [9]. Among the different types of inflammasomes, NLRP3 is activated by a broad spectrum of stimuli, including pathogens, damaged or dead cells, and toxic compounds [17, 65].

Specifically, ROS are known to be causative factors inducing NLRP3 activation. Indeed, the excessive production of ROS can lead to mitochondrial disintegration, resulting in NLRP3 inflammasome aberrant activation [66–69]. Moreover, ROS can oxidize mitochondrial DNA, including intracellular oxidized DNA and proteins aggregates/abducts (DAMPSs and/or PAMPs), thereby prompting the activation of NLRP3-mediated inflammasome inflammatory cell death [70]. Finally, ROS accumulation can trigger abnormal stress responses leading to redox-active transcription activators, one of the key determinants for the induction of NLRP3-driven pathological signaling [71, 72]. Also, several recent reports emphasize that pro-inflammatory signals are requisite for NLRP3 inflammasome activation, and that the activation is inhibited in absence of ROS [65, 73, 74].

Our data demonstrate an increase in NLRP3 levels in the hippocampus of animals treated with styrene, suggesting a role for inflammasome activation in mediating the neurotoxic effect of this volatile compound. NLRP3 inflammasome activation relies on two pathways, the canonical and non-canonical pathways. The non-canonical pathway depends on caspase-11 and is mediated by caspase-4, caspase-5, and caspase-8, whereas the canonical pathway predominantly depends on caspase-1 [75]. However, it is known that apoptotic executioner caspases, including caspase-3 and −7, act upstream of both caspase-8 and NLRP3-induced IL-1β maturation and secretion [76]. Of note, we found increased caspase-3 levels in the hippocampus of treated mice, and this result supports our hypothesis that styrene-induced ROS production and mitochondrial damage can trigger inflammation through the NLRP3 inflammasome. Our data also demonstrated that the neurotoxic effect of styrene can induce an increased level of MyD88 in the hippocampus of exposed animals, which correlates with a parallel increase of inflammatory markers, including COX-2, a downstream molecule of MyD88 activation [77]. This result also supports data indicating the involvement of inflammasomes in styrene neurotoxicity, considering that it is known that the increased secretion of cytokines can be regulated by the TLRs/MyD88 pathway, an upstream signal for activating the NLRP3 inflammasome [78]. Moreover, MyD88 has been reported to be crucial for microglia activation and subsequent proinflammatory mediator release in response to damaging stimuli [79]. Consistently, we found increased MyD88 levels and microglial activation in the hippocampi of styrene-exposed animals.

Notably, the neurotoxic effects of styrene described in this study show molecular mechanisms similar to those observed in various neurodegenerative disorders, including Alzheimer’s disease (AD), Parkinson’s disease (PD), multiple sclerosis (MS), or Huntington’s disease (HD). Among these, AD is driven by the accumulation of beta-amyloid (Aβ) plaques, tau pathology, and inflammation, ultimately leading to hippocampal dysfunction and cognitive decline [80]. Inflammation plays a central role in AD progression, by promoting cytokine release and ROS production through the interplay between microglia and astrocytes [81]. Glial activation can further exacerbate Aβ accumulation via microglial activation [82], amplifying oxidative stress and inflammatory damage [83]. The critical role of inflammation in AD pathology has also been recently supported by the protective effects of ecdysterone, an anti-inflammatory and antioxidant molecule, which, when combined with high-intensity interval training, improves synaptic function and mitigates cognitive deficits in an AD animal model [84]. Similarly, the activation of the inflammatory cascade contributes to the progressive loss of dopaminergic neurons in PD [85, 86] and clinical and experimental evidence also supports the role of peripheral inflammation in HD development and progression [87].

Recent evidence also implicates NLRP3 inflammasome-driven neuroinflammation in the pathogenesis of AD, where it promotes both Aβ and tau pathology [88] and triggers the release of IL1β and IL-18 [89]. Furthermore, inhibiting NLRP3 has been shown to attenuate AD pathology in tau transgenic mice [90]. Moreover, elevated plasma NLRP3 levels have been found to be strongly related to PD progression [91], while inhibiting NLRP3 activation alleviates inflammation in a PD mouse model [92]. Likewise, increased serum levels of inflammasome-related and neuroinflammatory components, such as NLRP3, NF-κB, and caspase-1 have been found in patients with MS, a chronic inflammatory disease, during early stages of pathology [93], while inhibiting inflammasome signaling showed protective effects in animal model of MS [94]. Taken together, this evidence showing the impact of neuroinflammation and inflammasome activation in CNS diseases, highlights the potential neurological risks associated with the exposure to styrene.

Even though we collected several experimental evidence describing increased oxidative stress, inflammation, glial cell, and inflammasome activation, some limitations of the study regard the lack of direct evidence of cause-effect mechanisms linking ROS and microglia/astrocyte activation via the NLRP3 pathway. Further studies are needed to support our hypothesis of the primary role of the ROS-induced NLRP3 activation in mediating glial cell response and the inflammatory damage caused by styrene in the hippocampus.

Moreover, it has to be taken into account that we used a dose of styrene of 400 mg/kg, based on previous studies, including ours, demonstrating that styrene can induce a toxic damage in both peripheral and central nervous system structures when administered by gavage using this dosage [22–25]. However, it would be interesting in further studies to evaluate the dose-dependent toxic effect of styrene on cognitive functions, by testing different dosages and obtaining a dose–response curve.

Collectively, our data provide evidence of ROS-mediated inflammasome activation damage in styrene-induced neurotoxicity in the hippocampus, leading to synaptic damage and memory deficits. From a translational point of view, our results highlight the high risk of chemical exposure at the occupational level for developing cognitive symptoms and suggest the ROS-NLRP3 signaling pathway as a possible target for preventing cognitive decline in exposed workers.

Specifically, our findings pave the way for the development of new therapeutic strategies based on antioxidant and anti-inflammatory approaches to mitigate styrene-induced neurotoxicity. In this context, previous studies from our group demonstrated the efficacy of the antioxidant Q-ter in counteracting styrene-induced cochlear damage [21]. More recently, we also compared the otoprotective effects of the antioxidant rosmarinic acid and the anti-inflammatory drug anakinra in suppressing NLRP3 activation in a model of styrene-induced ototoxicity (unpublished data).

Extending the evaluation of the protective effect of these antioxidant and anti-inflammatory compounds, as well as targeting directly NLRP3 inhibitors, may represent a promising avenue for developing effective therapeutic strategies to counteract the neurotoxic effects of styrene, particularly in occupational settings.

## Supplementary Information

Below is the link to the electronic supplementary material.

ESM 1 Supplementary Material 1 (DOCX 3.96 MB)

ESM 2 Supplementary Material 2 (DOCX 39.9 KB)

## Data Availability

Data is provided within the manuscript or supplementary information files.
