# Supplementary material for: Chronic Styrene Exposure Causes Oxidative Stress, Neuroinflammation, and Hippocampal Memory Dysfunction via NLRP3 Inflammasome Activation
Source: Mol Neurobiol. 2025 Dec 1;63(1):233. doi: 10.1007/s12035-025-05472-6 (PMC12669270; doi:10.1007/s12035-025-05472-6)
Supplement: Supplementary file 1 — Supplementary Material 1 (DOCX 3.96 MB) [file 12035_2025_5472_MOESM1_ESM.docx]

**Supplementary Figure 1**


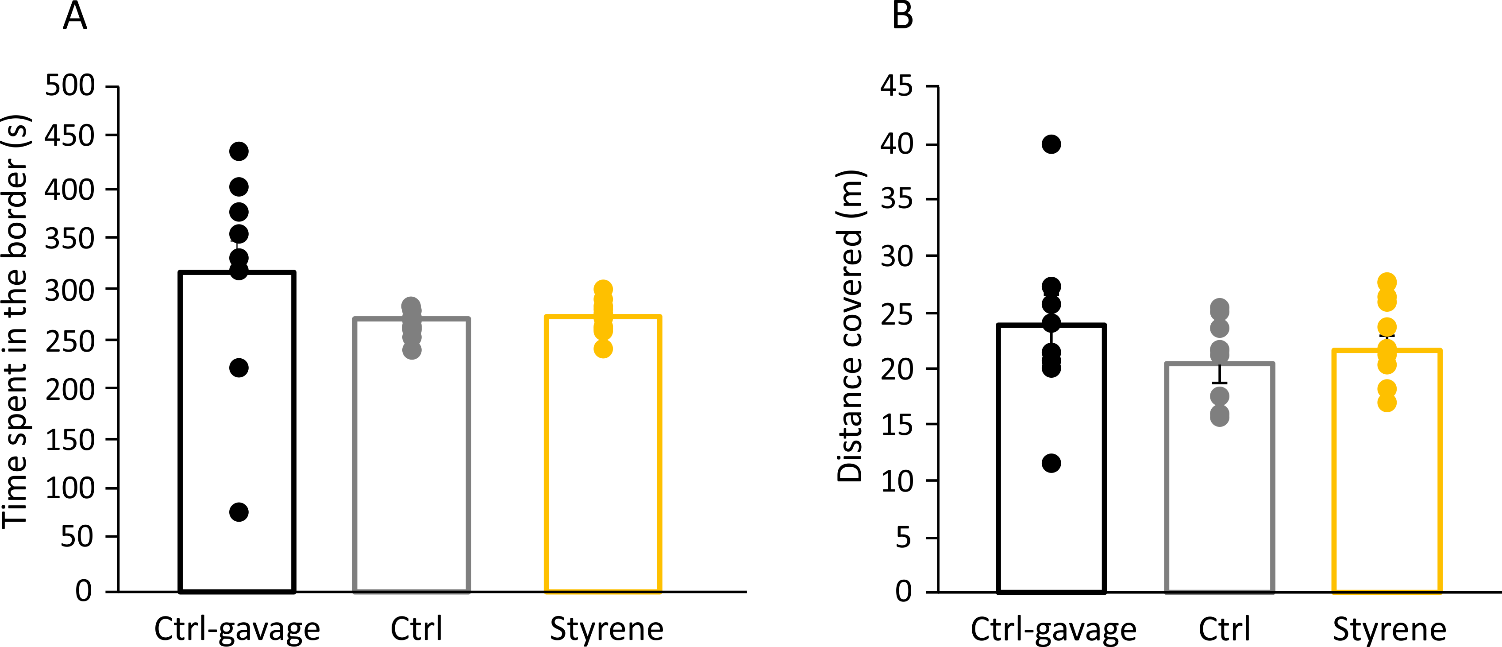


**Fig. S1. Anxiety and locomotor activity evaluations.**

Graphs showing the time spent in the border of the NOR open field arena (A) as a measure of anxiety or stress-related behavior, and the total distance covered in the NOR arena (B) as a measure of locomotor activity in Styrene, Ctrl and Ctrl-gavage groups (B: Ctrl n = 9, Ctrl-gavege n = 10, Styrene n = 10). No significant differences were found between groups. Data are expressed as mean ± SEM.

**
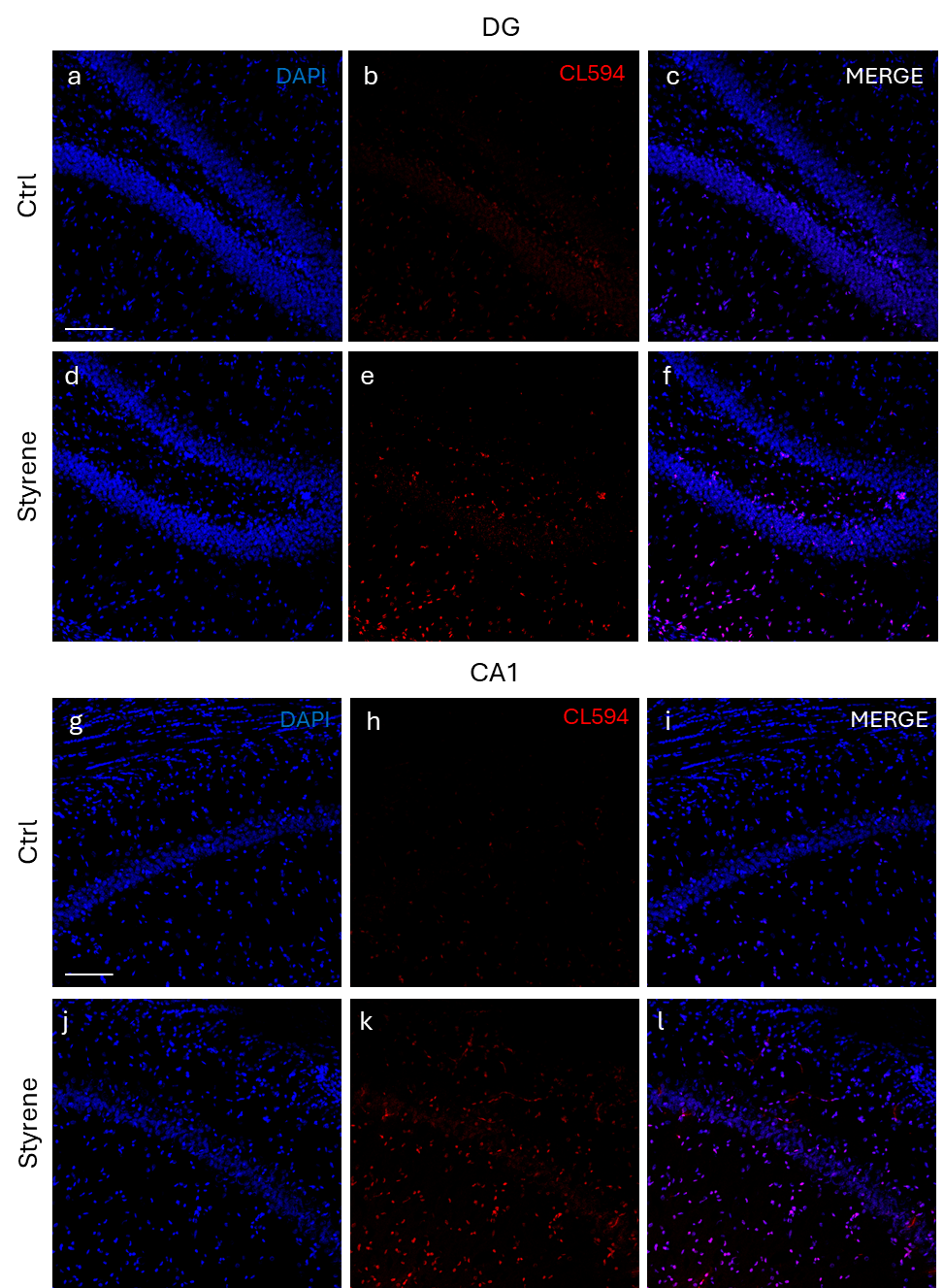
Supplementary Figure 2**

**Fig. S2. TUNEL Assay revealed apoptotic cells in the hippocampus of styrene treated- animals.**

Representative confocal images showing dentate gyrus (DG, a-f) and CA1 region (g-l) of the hippocampus of Ctrl and Styrene animals stained with DAPI (a,d,g,j) and with CL594 to detect apoptotic cells (b,e,h,k). Merged images (c,f,i,l) showed juxtaposed blue and red fluorescence. Scale bar: a and g, 100 μm

**
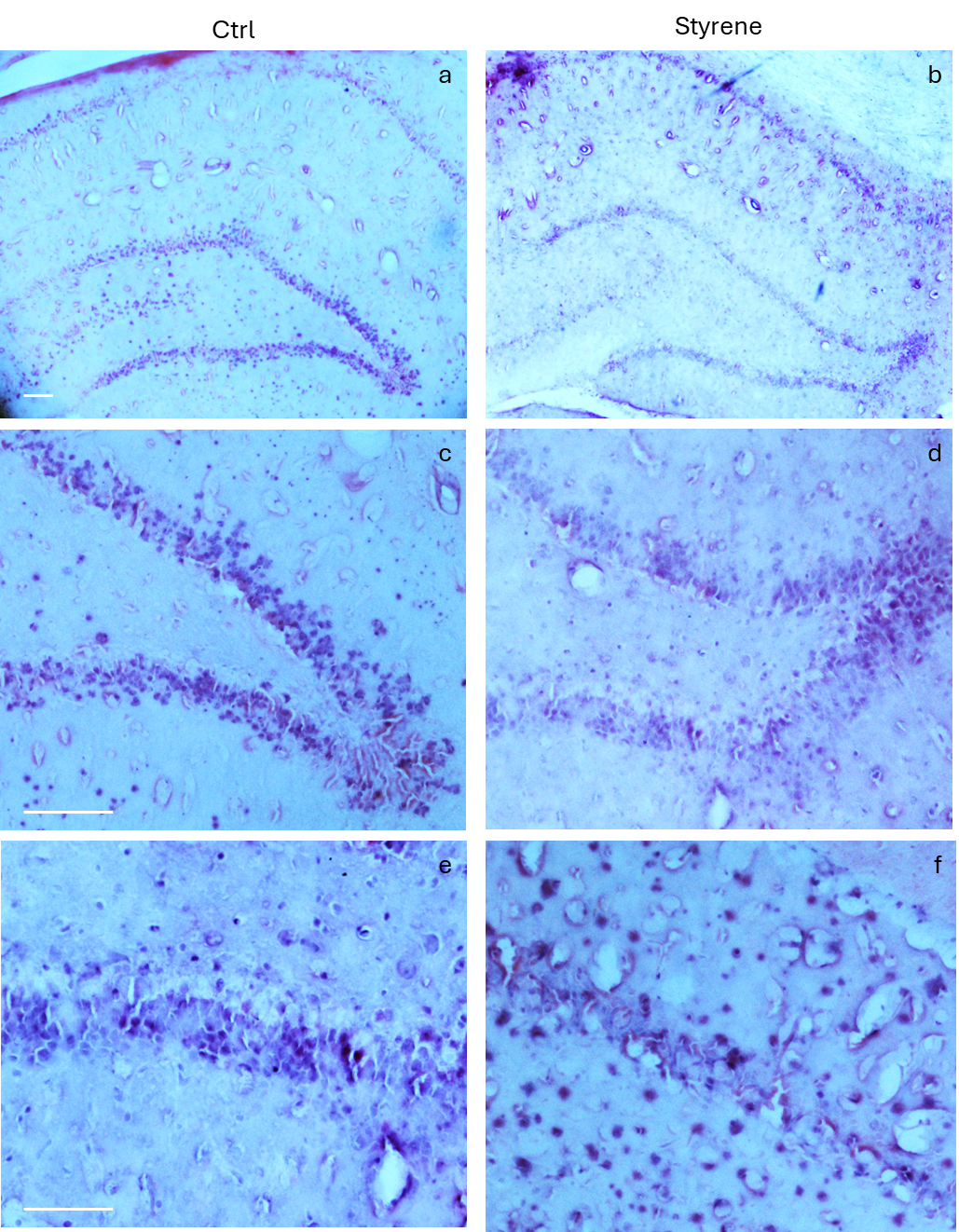
Supplementary Figure 3**

**Fig. S3. Morphological alterations in the hippocampus of styrene-treated animals.**

a-b: Representative images of brain sections containing the hippocampus in Ctrl and Styrene groups. Scale bar: 100 μm. c-f: higher magnifications of dentate gyrus (c-d) and CA1 (e-f) hippocampal regions showing disorganization, vacuolation, areas of cell loss and decreased thickness of cell layers in styrene-treated samples. Scale bar: 100 μm
