# Supplementary material for: Chronic Styrene Exposure Causes Oxidative Stress, Neuroinflammation, and Hippocampal Memory Dysfunction via NLRP3 Inflammasome Activation
Source: Mol Neurobiol. 2025 Dec 1;63(1):233. doi: 10.1007/s12035-025-05472-6 (PMC12669270; doi:10.1007/s12035-025-05472-6)
Supplement: Supplementary file 2 — Supplementary Material 2 (DOCX 39.9 KB) [file 12035_2025_5472_MOESM2_ESM.docx]

**Table S1.** List of reagents and antibodies used.

| Reagent type | Designation | Source | Identifiers | Additional information |
| --- | --- | --- | --- | --- |
| Antibody | Anti-IBA1(rabbit monoclonal) | Cell Signalign | Cat. No. #17198 | IF (1:100) |
| Antibody | Anti-rabbit IgG  AlexaFluor 488 (rabbit polyclonal) | Thermo Fisher Scientific | Cat.No. #A-21206 | IF (1:400) |
| Antibody | Anti-mouse IgG  AlexaFluor 546 (mouse polyclonal) | Thermo Fisher Scientific | Cat.No. #A10036 | IF (1:400) |
| Antibody | Anti-goat IgG  AlexaFluor 488 (mouse polyclonal) | Thermo Fisher Scientific | Cat.No. #A32814 | IF (1:400) |
| Nuclear Counterstains | DAPI | Thermo Fisher Scientific | Cat.No. D1306 | IF (0.5 mg/mL) |
| Antibody | Anti-GFAP (mouse monoclonal) | Cell Signalign | Cat. No. #3670 | IF (1:300)  WB (1:500) |
| Antibody | Anti-CD68 (mouse monoclonal) | Abcam | Cat. No. ab955 | WB (1:1000) |
| Antibody | Anti-COX-2 (rabbit monoclonal) | Cell Signalign | Cat. No. #12282 | WB (1:1000) |
| Antibody | Anti- cleaved-caspase 3 (rabbit polyclonal) | Millipore | Cat. No. #AB3623 | WB (1:100) |
| Antibody | Anti- Caspase-3 (rabbit polyclonal) | Santa Cruz Biotechnology | Cat. No. sc-7272 | WB (1:1000) |
| Antibody | Anti-NLRP3 (rabbit monoclonal) | Invitrogen | Cat. No.  MA5-32255 | WB (1:1000) |
| Antibody | Anti-TNF-α  (mouse monoclonal) | Santa Cruz Biotechnology | Cat. No. sc-52746 | WB (1:1000) |
| Antibody | Anti- IL-1β (rabbit polyclonal) | Santa Cruz Biotechnology | Cat. No. sc-7884 | WB (1:1000) |
| Antibody | Anti-Nitro tyrosine  (rabbit) | Cell Signalign | Cat. No. #06-284 | WB (1:1000) |
| Antibody | Anti-4HNE (rabbit polyclonal) | Alpha Diagnostic International | Cat. No. #HNE11-S | WB (1:1000) |
| Antibody | Anti-GAPDH  (mouse monoclonal) | Abcam | Cat. No. ab8245 | WB (1:10000) |
| Antibody | Anti-β-Actin (rabbit monoclonal) | Sigma-Aldrich | Cat. No.  SAB5600204 | WB (1:1000) |
| Antibody | Anti-VGlut1  (rabbit monoclonal) | Cell Signaling | Cat. No. #47181 | WB (1:1000) |
| Antibody | Anti-Gephyrin  (rabbit monoclonal) | Abcam | Cat. No. ab177154 | WB (1:1000) |
| Antibody | Anti-MyD88 (rabbit polyclonal) | Cell Signaling | Cat. No. #3699 | WB (1:1000) |
| Antibody | Anti-HO-1 (mouse monoclonal) | Abcam | Cat. No. Ab13248 | WB (1:1000) |
| Antibody | Anti-Phospho-CaMKII (Thr286) (rabbit monoclonal) | Cell Signaling | Cat. No. #12716 | WB (1:1000) |
| Antibody | Anti-CaMKII (rabbit monoclonal) | Invitrogen | Cat. No.  #MA5-42588 | WB (1:1000) |
| Antibody | Anti-Mn-SOD  (rabbit polyclonal) | Millipore | Cat. No. #06-984 | WB (1:1000) |
| Antibody | Anti-8-isoprostane  (goat polyclonal) | Oxford Biomedical Research | Cat. No. IS20 | IF (1:100) |
| Commercial Kit | H&E Staining Kit (Hematoxylin and Eosin) | Abcam | Cat. No. ab245880 |  |
| Commercial Kit | Coralite 594 TUNEL Assay Apoptosis Detection Kit | Proteintech | Cat. No. PF00009 |  |
